# Explainable Reinforcement Learning for the Initial Design Optimization of Compressors Inspired by the Black-Winged Kite

**DOI:** 10.3390/biomimetics10080497

**Published:** 2025-07-29

**Authors:** Mingming Zhang, Zhuang Miao, Xi Nan, Ning Ma, Ruoyang Liu

**Affiliations:** 1 School of Mathematics Statistics and Mechanics, Beijing University of Technology, Beijing 100124, China; mmzhang@bjut.edu.cn; 2 Aero Engine Academy of China, Beijing 101304, China; Nanxi_UT@outlook.com (X.N.); maning216@163.com (N.M.); ruoyang_liu@foxmail.com (R.L.)

**Keywords:** reinforcement learning, compressor initial design, explainability, decision tree, black-winged kite algorithm, rule extraction

## Abstract

Although artificial intelligence methods such as reinforcement learning (RL) show potential in optimizing the design of compressors, there are still two major challenges remaining: limited design variables and insufficient model explainability. For the initial design of compressors, this paper proposes a technical approach that incorporates deep reinforcement learning and decision tree distillation to enhance both the optimization capability and explainability. First, a pre-selection platform for the initial design scheme of the compressors is constructed based on the Deep Deterministic Policy Gradient (DDPG) algorithm. The optimization space is significantly enlarged by expanding the co-design of 25 key variables (e.g., the inlet airflow angle, the reaction, the load coefficient, etc.). Then, the initial design of six-stage axial compressors is successfully completed, with the axial efficiency increasing to 84.65% at the design speed and the surge margin extending to 10.75%. The design scheme is closer to the actual needs of engineering. Secondly, Shapley Additive Explanations (SHAP) analysis is utilized to reveal the influence of the mechanism of the key design parameters on the performance of the compressors in order to enhance the model explainability. Finally, the decision tree inspired by the black-winged kite (BKA) algorithm takes the interpretable design rules and transforms the data-driven intelligent optimization into explicit engineering experience. Through experimental validation, this method significantly improves the transparency of the design process while maintaining the high performance of the DDPG algorithm. The extracted design rules not only have clear physical meanings but also can effectively guide the initial design of the compressors, providing a new idea with both optimization capability and explainability for its intelligent design.

## 1. Introduction

The aero-engine is known as the heart of the airplane. As a key component of the aero-engine, the compressor is the technical bottleneck of aero-engine development. High-performance compressors are often required in order to reach high efficiency, a high pressure ratio, and a high surge margin. But, in the actual engineering design, these three indicators often cannot be satisfied at the same time. A large number of coupled design parameters and conflicting performance requirements have brought great difficulty to the design of compressors. In order to meet the performance requirements of the compressors, there is an urgent need to explore new methods and technologies to ensure the aerodynamic design of axial flow compressors and to improve the aerodynamic design capability and level of the compressors. At present, the design system of the compressors includes a one-dimensional initial design, a through-flow design, a two-dimensional leaf design, and a three-dimensional design. The one-dimensional initial design, as the first link in the aerodynamic design process, is of great significance for the screening of design solutions from the overall performance of the compressor. With the development of machine learning and deep learning, exploring the use of artificial intelligence methods for the initial design of the compressors has become an inevitable path in view of the performance enhancement challenges faced in the design process of axial flow compressors at this stage.

Artificial intelligence and machine learning methods involve several research areas such as problem solving, expert systems, machine learning, pattern recognition, etc., which provide new ideas for traditional initial design techniques for compressors. Ransom et al. [1] constructed a numerical library using all known initial successful design solutions for industrial compressors. Used as training data for machine learning models, it is able to provide reasonable initial design solutions for compressors. Bourabia et al. [2] proposed an initial design method for centrifugal compressors based on the coupling of an optimization algorithm and a one-dimensional streamlined model, which is capable of generating optimal design solutions. Ma et al. [3] proposed a deep learning method based on deviation angle prediction and embedded into a one-dimensional computational program, which replaced the original deviation angle model, achieving a higher accuracy prediction. For the same problem, Yan et al. [4] used a genetic algorithm with comparison to obtain the computational coefficients of the deviation angle, work, and efficiency in the empirical model. An eight-stage compressor was optimized and validated using the one-dimensional results. These methods, while realizing performance improvements, strongly rely on fixed data. They do not cope well with environmental changes and lack the perspective to accumulate design experience.

Reinforcement learning allows the user to acquire knowledge through interactions with the environment and to learn optimal policies autonomously [5,6]. It can also be applied to various types of engineering design with the advantage of experience accumulation without a priori knowledge. Dworschak et al. [7] explored the feasibility of reinforcement learning in the design of engineering automation. Sabzevari et al. [8] utilized multi-agent reinforcement learning to adjust the levels of metabolic enzymes, thereby increasing the production of microbial strains. Brown et al. [9] used reinforcement learning to automate the design of 2D discretized topologies. In the design of aero-engines, Qin et al. [10] proposed a multi-objective optimization on the cascade blade profile using reinforcement learning algorithms. The optimization results were obtained in terms of the total pressure loss and laminar flow area. Xu et al. [11] proposed an improved Deep Deterministic Policy Gradient algorithm for compressor design and trained multiple agents to improve the performance of 3D transonic rotors. Song et al. [12] proposed a multi-objective acoustic sensor arrangement optimization method based on the results from information field fitting and reinforcement learning optimization. This verified the advantages of the proposed method in terms of optimal sensor arrangement and crack detection. Zhang et al. [13] proposed a deep-reinforcement-learning-based active surge control method to ensure the stability of the compressor over a wide operating range. Liu [14] conducted a one-dimensional aerodynamic optimization design of a nine-stage axial flow compressor using the DDPG algorithm, which significantly improved the efficiency and margin of the compressor. Other than what has been mentioned, reinforcement learning has been studied less frequently in the initial design of compressors.

There are two key limitations in the existing research. On the one hand, the range of optimization design variables is relatively limited, making it difficult to fully reflect the complex requirements in engineering practice. On the other hand, despite the encouraging performance of reinforcement learning in compressor design, it still faces challenges in practical applications. These “black-box” models lack interpretability and are unable to solidify complex design experience into reusable design guidelines.

When involving complex data information, it is crucial to gain insight into the decision-making process of machine learning. Although it can be successfully modeled using black-box learning algorithms, any modification to the optimization problem may have far-reaching consequences. The model interpretability has received increasing attention. To overcome these weaknesses, interpretable reinforcement learning has emerged as a promising approach. Shapley Additive Explanations (SHAP) is an interpretable artificial intelligence technique derived from game theory for quantifying the marginal contribution of each feature to the model output [15]. The ranking of SHAP values is the importance ranking of features. According to the importance ranking, it can capture the key features to understand the degree of influence on the output. Decision trees are another typical example of interpretable models. Classification and regression trees (CARTs) have achieved advanced performance on a wide range of prediction problems and gained applications in rule extraction [16]. Juan de Ona et al. [17] used a decision tree to extract decision rules from traffic accident reports for road safety analysis. Zhang et al. [18] used decision trees to study the correlation rules between alarms and faults of synchronous digital system optical communication devices in power systems, providing an effective solution of intelligent analysis. These tree models are usually not used for reinforcement learning because they cannot be updated online by stochastic gradient descent. Andrew Silva et al. [19] overcame this limitation by allowing gradient updating of the entire tree, thus providing interpretable policy extraction for reinforcement learning. Youri Coppens et al. [20] benchmarked deep neural network RL policies from Mario AI to distill a soft decision tree to achieve an explanation of policy learning behavior. Zhu et al. [21] used an improved decision tree to extract rules from a deep reinforcement learning approach for solving a traffic signal control problem. However, in the field of aero-engine compressor design, the research related to this method is still lacking.

To address the above challenges, this paper uses explainable reinforcement learning via policy extraction, guided by the DDPG-SHAP policy, to learn decision tree policies and extract rules for compressor design. In addition, due to the splitting limitation, it can obtain more concise rules, which can improve the explainability of the design rules. Through experimental tests, it verifies the effectiveness of the design rules and finds the balance between explainability and performance.

The main contributions of this paper are:

(1) This study significantly expands the optimization space by introducing more key design variables. Compared with the limited parameters usually focused on in the existing work, this method can simultaneously deal with 25 multi-dimensional design variables, including inlet airflow angle, tip diameter, load factor, etc. It makes the initial design of compressor more flexible, comprehensive, and closer to the actual needs of complex engineering.
(2) The decision tree distillation technique is firstly introduced into the compressor initial design to enhance the design explainability. The improved DDPG algorithm is used to optimize the initial parameters. The key design features are revealed by SHAP value analysis. And the explainable design rules are extracted by the decision tree inspired by black-winged kite. This new paradigm of “Intelligent Optimization-Rule Extraction” not only optimizes the performance of compressor but also transforms the data-driven intelligent optimization into explainable engineering experience. This application perspective provides a new thinking for compressor design that combines high performance and transparency.

The main contents of this paper are arranged as follows. Firstly, the basic theory of reinforcement learning and algorithm improvement are introduced in detail. Secondly, the relevant theories of the two explainable methods, SHAP and decision tree, are presented. Then, the pre-screening platform for initial design solutions of compressor based on DDPG algorithm is elaborated in detail with the optimization results. Subsequently, the SHAP explainability analysis is carried out on the screened design schemes, while the initial design rules of compressors based on the decision tree distillation method are introduced in detail. Finally, in order to validate the effectiveness of the extracted design rules, experiments are tested in the same context. The validation results show that the design rules extracted from the decision tree retain the good performance of the reinforcement learning network, while substantially improving in terms of transparency and explainability.

## 2. Reinforcement Learning Architecture

Reinforcement learning is a learning method in which an agent interacts with its environment in order to accumulate experience and obtain feedback rewards. Markov decision process [22] is its theoretical foundation, which can be described by a tuple <S,A,p,R,γ>. *S* denotes the state space, A denotes the action space, and p is the state transfer probability function, which represents the probability that the environment transfers to $s^{\prime }$ in the current state s after the action *a* of the intelligent agent. And its mathematical representation is as follows:

(1) $$\left.\begin{array}{c} S\times A\to \left[0,1\right]\\ p\left(s^{\prime }|s,a\right)=Pr\left\{\left(S_{t+1}=s^{\prime }|S_{t}=s,A_{t}=a\right)\right\} \end{array}\right\}$$

R is the reward function, which represents the reward value fed back to the agent by the environment. γϵ[0,1] is the discount factor, which is used to calculate the cumulative discounted reward. The goal of reinforcement learning is to find an optimal policy $\pi^{*}$ to maximize the desired reward.

Reinforcement learning algorithms can be categorized into two types depending on the state and action space: value-based policies and probability-based policies. The former is used to deal with the case where both states and actions are discrete values, selecting the action with the highest value at the moment with a simpler structure. The latter is used to deal with continuous optimization problems, usually with the help of neural networks in a complex structure. The initial design problem for axial flow compressor is a complex continuous optimization problem and, hence, the DDPG algorithm [23] is used in this paper.

### 2.1. Deep Deterministic Policy Gradient

The DDPG algorithm is a continuous action space optimization algorithm using deep neural networks to learn approximate action values and approximate deterministic policies with a dual-network architecture. It is able to efficiently find near-global optimal parameter combinations in a high-dimensional parameter space.

The DDPG algorithm adopts a deterministic policy based on deep neural networks, and the main principles of the DDPG algorithm include the actor-critic framework, the dual-network architecture, the noise exploration mechanism, and the soft update mechanism of the target network parameters. The following is a detailed description of each part.

(1) Actor–Critic

The Actor–Critic (AC) policy gradient method combines the ideas of the value function approximation method and the policy gradient method. The algorithm includes a policy network and a value network. The policy network is responsible for updating the policy parameters and plays the role of an actor. The value network is responsible for calculating the action values and plays the role of a critic.

**Figure 1:**
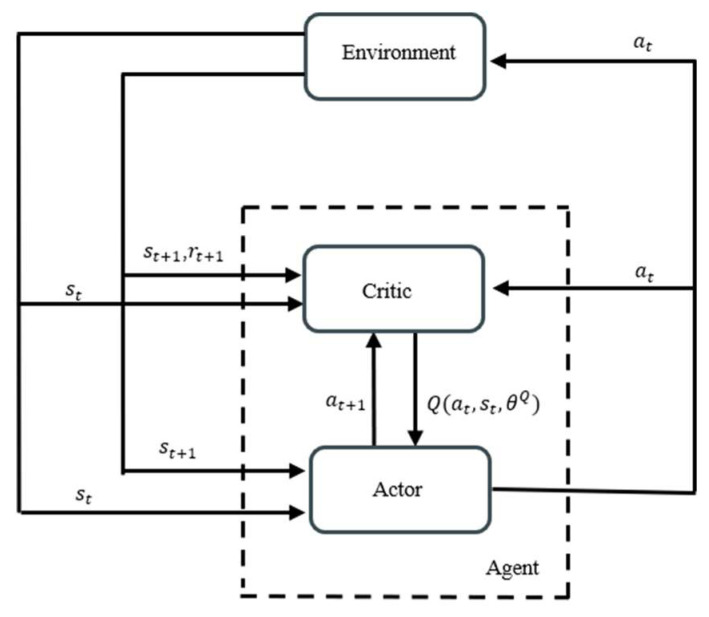
Schematic of the actor–critic policy gradient method.

As shown in Figure 1, a complete iteration of the actor–critic policy gradient method consists of (i) the agent perceiving the environment state $s_{t}$; (ii) the policy network selecting the action $a_{t}$ to be performed according to the current policy, imposing the action on the environment, shifting the environment state to $s_{t+1}$, and feeding back the immediate reward $r_{t+1}$; (iii) the agent perceiving the environment state $s_{t+1}$, selecting the action according to the current policy, and passing the action to the value network; (iv) the value network calculating two action values $Q(s_{t},a_{t},\theta^{Q})$ and $Q(s_{t+1},a_{t+1},\theta^{Q})$ based on $\left(s_{t},a_{t}\right)$ and $\left(s_{t+1},a_{t+1}\right),$ respectively, and transmitting $Q(s_{t},a_{t},\theta^{Q})$ to the policy network; and (v) the policy network and the value network updating their own parameters, respectively.

(2) Dual-network architecture

The DDPG consists of four networks as the predictive policy network, the target policy network, the predictive value network, and the target value network. The structures of the prediction network and the target network are identical. The parameters of the prediction network are updated with training. The target network does not participate in training, and its parameters are periodically copied from the prediction network in a soft-update fashion.

In the DDPG algorithm, the optimization objective of the policy network is defined as the cumulative discount reward,

(2) $$J\left(\theta^{\mu }\right)=E_{\theta^{\mu }}[r_{0}+\gamma r_{1}+\gamma^{2}r_{2}+\cdots ]$$

The optimization deterministic policy function is namely the maximization objective function $J(\theta^{\mu })$ [24].

The value network uses the squared error based on the TD difference as the loss function. The process of optimizing the value network is to minimize the loss function ${min}_{\theta^{Q}}L(\theta^{Q})$.

(3) $$L\left(\theta^{Q}\right)=E[{\left(Q\left(s,a\right)-\hat{Q}(s,a;\theta^{Q})\right)}^{2}].$$

(3) Noise exploration

DDPG implements global exploration by adding noise to the action space. It means adding noise to the computed action as

(4) $$a_{t}=\hat{\pi }(s_{t};\theta^{\mu })+N_{t}$$

(4) Soft update for target network parameters

DDPG uses a soft update method. After the predictive network parameters are updated each time, the target network parameters are brought closer to the predictive network to some extent. The update formula is shown as

(5) $$\left\{\begin{array}{c} {\theta^{Q}}^{\prime }\leftarrow \tau \theta^{Q}+(1-\tau ){\theta^{Q}}^{\prime }\\ {\theta^{\mu }}^{\prime }\leftarrow \tau \theta^{\mu }+(1-\tau ){\theta^{\mu }}^{\prime } \end{array}\right.$$

where τ is a hyperparameter much smaller than 1.

### 2.2. Prioritized Experience Replay

Experience replay is a key technique to enable the organic integration of deep learning and reinforcement learning. Experience replay refers to storing historical experience data in an experience replay pool, which can be used as the training data of the value network. By randomly sampling from the experience replay pool, the temporal correlation between the data is greatly reduced and the performance of the algorithm is improved. However, the traditional experience replay takes a uniform sampling approach, where each sample is sampled with equal probability. Although this sampling approach ensures sample diversity, the learning efficiency of the model is not high. In the learning process, instead of uniformly replaying all experiences, frequently replaying experiences associated with more successful attempts can help to better recognize the consequences of misbehavior and improve learning efficiency. Thus, Tom Schaul et al. [25] proposed a prioritized experience replay technique.

The sample priority is defined as follows,

(6) $$\left|\delta_{i}\right|=\left|y_{i}-\hat{Q}(s_{i},a_{i};\theta )\right|$$

The definition of priority should satisfy two conditions. Firstly, the priority should be numerically monotonically increasing with the absolute value of the error. This is to satisfy the fact that the sample with larger absolute value of the error, i.e., the sample with larger priority, obtains a greater chance to be sampled. Secondly, the value of the priority should be greater than 0, that is, the sampling probability is larger than 0.

The two basic principles of sampling by the prioritized experience replay mechanism are as follows:

(1) The probability of a sample being sampled should be positively correlated with the sample priority.
(2) Each sample should have a chance to be sampled, i.e., the probability of being sampled is greater than 0.

The probability of a sample being sampled is defined as

(7) $$P\left(i\right)=\frac{\delta_{i}^{\alpha }}{\sum_{k}p_{k}^{\alpha }}$$

where $\delta_{i}$ > 0 is the priority of the sample, and the exponent α∈[0,1]  determines the degree of the priority.

Since the distribution of the data obtained according to the priority sampling and the overall distribution of the data in the original replay pool are different, they have different expectations. This bias will have an impact on the convergence of the training process, and IS weights are introduced to correct the bias. IS weights are defined as

(8) $$\omega_{i}^{\prime }={\left(\frac{1}{N}\cdot \frac{1}{P\left(i\right)}\right)}^{\beta }$$

where β∈[0,1] is the compensation coefficient, N is the capacity of the empirical replay pool, and $P\left(i\right)$ is the probability of the sample being sampled.

**Figure 2:**
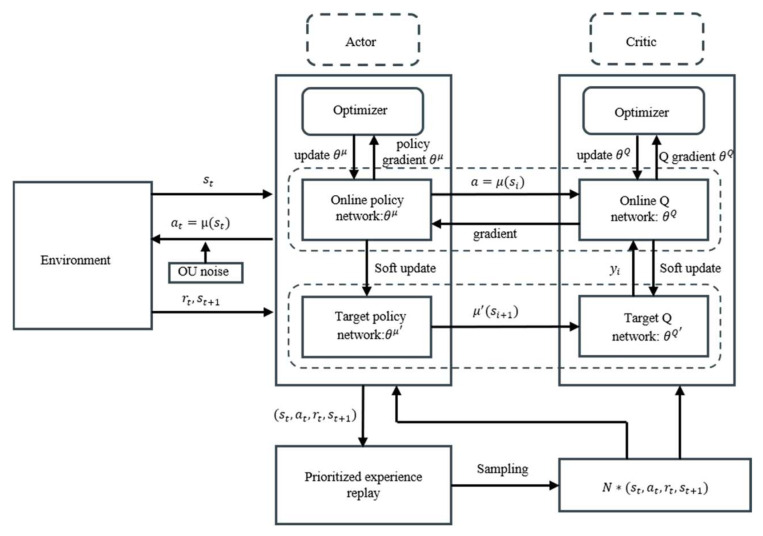
Prioritized experience replay DDPG architecture.

Hou et al. [26] improved the DDPG algorithm with a prioritized experience replay mechanism. This paper follows the logic of improved DDPG algorithm with the architecture of the improved DDPG shown in Figure 2.

## 3. Explainability for Intelligent Design

### 3.1. SHAP Analysis

The core idea of SHAP is to calculate the marginal contribution of each feature to the model output. It generates a Shapely value for each input feature, which assigns an importance score to each part of the input [15]. This value indicates the contribution of the feature to the prediction of the specified data. Some factors positively affect the prediction probability, while others negatively affect it.

The SHAP value is calculated as

(9) $$\Phi =\Phi_{0}+\sum_{i=1}^{n}\Phi_{i}$$

where $\Phi_{0}$ is the average prediction value of all the samples in the model and $\Phi_{i}$ is the SHAP value of the feature i, which reflects the marginal contribution of the feature to the prediction result.

SHAP is nowadays considered a central contribution to the field of explainable AI [27] and has evolved in different versions in ongoing research for image inputs [28] and textual inputs [29] or alternatively for Random Forest Models [15]. SHAP disassembles complex model predictions into comprehensible feature contributions and is theoretically complete and intuitive. Despite challenges such as computational costs, its balance in terms of explainability, fairness, and utility makes it useful in the field of explanation.

### 3.2. Decision Tree

#### 3.2.1. Fundamental Theory

Decision tree is a supervised learning algorithm based on tree structure, which is suitable for nonlinear data with intuitive explainability and computation efficiency.

The decision principle of decision trees is to recursively divide the feature space into non-overlapping regions and make predictions within each region. The node splits of the decision tree are based on binary splits using the values of the features. Each node split is independently and greedily selected, with each child node reevaluating all the available features and considering only the optimal split for the current node. This design allows the decision tree to capture the nonlinear effects of features and adapt to local feature importance changes.

Compressor design rule extraction is a typical continuous feature and belongs to the regression prediction problem. CART is an advanced decision tree model for solving regression problems [30]. The splitting objective of the nodes of the CART regression tree is to minimize the mean-square error to find splitting points that minimize the variance in the target values of the child nodes.

If $y_{i}$ denotes the output of the training set $S=\{\left(x_{1},y_{1}\right),\left(x_{2},y_{2}\right),\cdots ,(x_{n},y_{n})\},$ $f(x_{i})$ is the predicted value, and the prediction error is denoted as $\sum_{x_{i}\in S}{\left[y_{i}-f(x_{i})\right]}^{2}$. The goal of the fit is to seek the best division point in the features and find every $f(x_{i})$ that minimizes the sum of squared errors.

Applied to the CART regression tree, the mathematical expression is as follows:

(10) $$\underset{j,k}{\min}[\underset{C_{1}}{\min}\sum_{x_{i}\in R_{1}\left(j,k\right)}{\left(y_{i}-C_{1}\right)}^{2}+\underset{C_{2}}{\min}\sum_{x_{i}\in R_{2}\left(j,k\right)}{\left(y_{i}-C_{2}\right)}^{2}],$$

where j,k denotes the division point k of the *jth* variable, $R_{1}(j,k)$ denotes the left region under the division, $R_{2}(j,k)$ denotes the right region under the division, and $C_{1},C_{2}$ are the optimal output values for regions $R_{1}\left(j,k\right)$ and $R_{2}\left(j,k\right)$.

The decision process of the decision tree is as follows. When a new sample enters the tree, it starts from the root node and judges towards the left/right child node based on the eigenvalues, repeating the judgment until it reaches the leaf node. It outputs the average of the target values of all the training samples as the predicted value.

#### 3.2.2. Decision Tree Tuning by BKA

With the continuous development of meta-heuristic algorithms, more accurate optimization solutions for complex engineering problems have been widely used. Leonardo [31] proposed a hybrid multi-population meta-heuristic algorithm combining crow search algorithm and symbiotic organisms search for solving the load-sharing optimization problem. Sattar [32] proposed a new type of nature-inspired optimization algorithm, called Smart Flower Optimization Algorithm (SFOA). SFOA successfully solved four different engineering design problems (three-bar truss, tension/compression spring, speed reducer, and welded beam) and the results demonstrated the ability to find an optimal solution. Zhang [33] applied the grey wolf optimization algorithm for parameter optimization of a support vector model to achieve the prediction of stall inception in compressor. In order to better deal with complex problems, Wang [34] developed a new meta-heuristic algorithm called black-winged kite (BKA). The algorithm has a strong evolutionary ability with fast search speed and high optimization finding ability, and it is gradually used in engineering optimization problems.

In this section, BKA performs automatic parameter search for the combination of maximum depth and minimum number of samples of leaf nodes in a decision tree model. The black-winged kite optimization algorithm is a new population intelligence optimization inspired by black-winged kite migratory and predatory behaviors, which has strong evolutionary ability, fast search speed, and integrates the Cauchy’s variation strategy to enhance the global search ability. The algorithm flow is as follows:

Step 1: Initialize population individual positions.

Step 2: Calculate population individual fitness and select the best adapted individual as leader.

Step 3: Attack behavior and update individual positions,

(11) $$y_{t+1}^{i,j}=\left\{\begin{array}{c} y_{t}^{i,j}+n\left(1+\sin\left(r\right)\right)\times y_{t}^{i,j}\;\;\;\;p<r\\ y_{t}^{i,j}+n\left(2r-1\right)\times y_{t}^{i,j}\;\;\;\;\;\;\;\;else \end{array}\right.,$$

where $y_{t}^{i,j}$ represents the updated value of the $j^{th}$ dimensional position of the $i^{th}$ individual of the population at the $t^{th}$ iteration, $n=0.05\times e^{-2({\frac{t}{T})}^{2}}$, T is the total number of iteration rounds, r is a random number between 0 and 1, and p is a constant which is usually taken as 0.9.

Step 4: Migration behavior and update the leader’s position

(12) $$y_{t+1}^{i,j}=\left\{\begin{array}{c} y_{t}^{i,j}+C(0,1)\times (y_{t}^{i,j}-L_{t}^{j})\;\;\;\;\;\;\;\;\;\;F_{i}<F_{ri}\\ y_{t}^{i,j}+C(0,1)\times (L_{t}^{j}{-m\times y}_{t}^{i,j})\;\;\;\;\;\;\;\;\;else \end{array}\right.,$$

where C(0,1) represents the Cauchy variation, $L_{t}^{j}$ is the value of the $j^{th}$ dimensional position of the leader in the $t^{th}$ iteration, $F_{i}$ is the fitness of the current leader, $F_{ri}$ is the fitness of a random individual, and m=2×sin(r+π/2).

Step 5: Calculate the new population fitness.

Step 6: Determine whether the termination condition is satisfied; if it is satisfied, output the optimal solution; if it is not satisfied, repeat step 2 to step 5.

**Figure 3:**
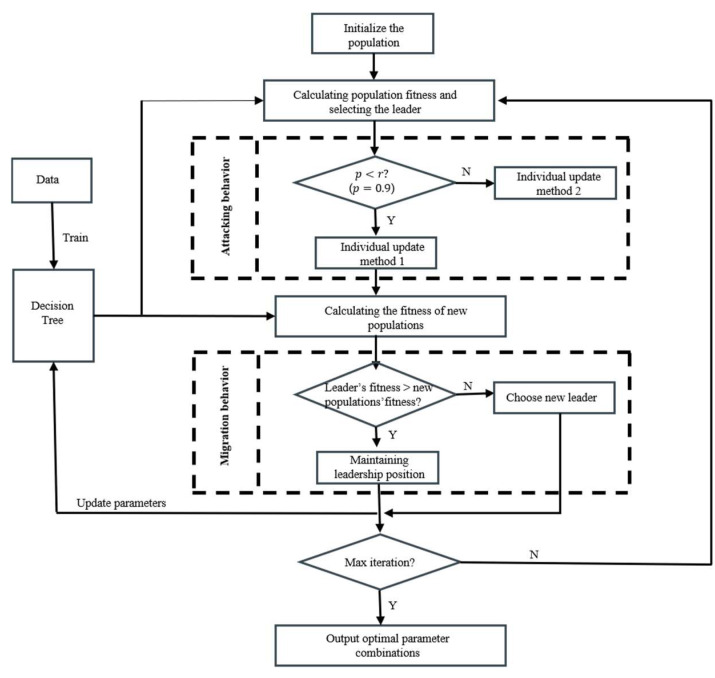
Black-winged kite algorithm for automatic parameter seeking in decision trees.

The population individual is defined as a two-dimensional vector $X=[x_{1},x_{2}]$. $x_{1},x_{2}$ represent the maximum depth of the decision tree and the minimum number of samples of the leaf nodes, respectively. And the size of the population is 50, with a maximum number of iterations of 100. The mean-square error is chosen as the fitness. The flowchart of the optimization process of the decision tree is as follows, inspired by the black-winged kite optimization algorithm, in Figure 3.

## 4. Filtering Platform on Initial Design Options for Compressor

The subject of this paper is a six-stage axial compressor. The Reynolds number of this compressor at the ground design point is greater than $1\times 10^{6}$, which is within the Reynolds number self-modeling region, and the effect of Reynolds number on the performance of the compressor is negligible. The design pressure ratio of the compressors is 5.87 and the flow rate is 9.38 kg/s.

HARIKA is the 1D calculation program. It calculates the aerodynamic parameters, structural parameters, and stage characteristics of compressor according to the given design specifications. Zhong [35] validated the accuracy of HARIKA by comparing the results of HARIKA calculations with the experimental results of Stage 35. Chen [36] used the program to carry out a one-dimensional design of the compressor and completed a three-dimensional design of a compressor.

In order to realize the initial design of the compressor, the one-dimensional characteristic program HARIKA is combined with DDPG algorithm. It is also necessary to determine the state space, the action space, the rewards, and the environmental settings of the design problem.

### 4.1. Algorithm Settings

In the design optimization, efficiency and surge margin are selected as the optimization objectives. The basic settings of the DDPG algorithm are as follows:

(1) Environment. The HARIKA program is selected as the environment to interact with the agent. The inputs to HARIKA include design requirements and design variables. The design requirements include the given inlet total pressure, total temperature, pressure ratio, flow rate, etc. And the design variables include the tip diameter, rotational speed, and load coefficients, which need to be determined.
(2) States. According to the design requirements, the inlet airflow angle, the tip diameters of each stage, the inlet axial velocity of each stage rotor, the outlet axial velocity of each stage rotor, the load factors of each stage, the reaction, and the reaction increment are selected as the design variables, which affect the aerodynamic performance to a large extent. The definitions and the values of these parameters are shown in Table 1. The state of DDPG is set as a one-dimensional array of these 25 variables, and the value of each variable in the array is equal to the value of the corresponding design variable.
(3) Actions. The values of the 25 design variables are defined as actions, and the action array is the same size as the state array with 25 variables.
(4) Rewards. The compressor efficiency at the design speed can be obtained by calculating with the HARIKA program, as well as the surge margin. The formula for the surge margin is defined as,
  (13) $$SM=\left(\frac{\pi_{s}^{*}}{\pi_{o}^{*}}\cdot \frac{G_{o}}{G_{s}}-1\right)\times 100\%$$
  where *π* is the pressure ratio, *G* is the flow rate, the subscript *o* represents the operating point, and *s* represents the surge point.
  The D factor is a key parameter in the compressor aerodynamic design to measure the degree of diffusion of the airflow within the cascade. The larger the D factor, the more severe the deceleration of the airflow in the cascade. The boundary layer is prone to separating with a large D factor, resulting in a loss of efficiency or stalling. The DLK as the D factor of the rotor is used to assess the risk of separation of the suction surfaces of the rotor blades and directly affects the stall margin of the rotor. The DLA as the D factor of the stator is used to control the boundary layer development, which affects the secondary flow and total pressure loss in the endwall. In the design of a six-stage compressor, DLA is set as less than 0.57.
  During the design process, the target is to obtain the greatest possible efficiency and margin, with satisfying the constraints leading to the design of a multi-constraint reward function as
  (14) $$R=\omega_{1}*\eta +I_{1}*\omega_{2}*C_{1}+I_{2}*\omega_{3}*C_{2}$$
  where η is the efficiency and $C_{1},C_{2}$ are the two penalty terms related to the surge margin and the D-factor, respectively. $I_{1}$ and $I_{2}$ are the two indicator functions, which are only effective if the constraints are not satisfied. $\omega_{1},\omega_{2}$, and $\omega_{3}$ are the weighting coefficients.

**Figure 4:**
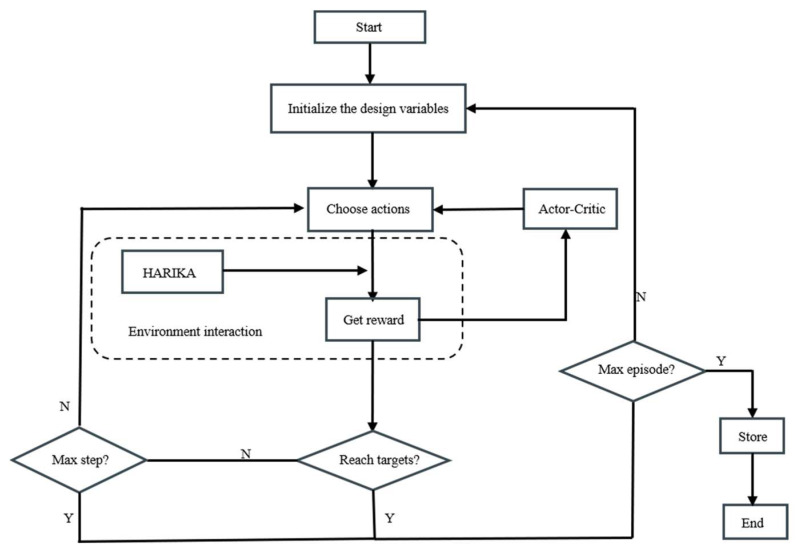
DDPG-based filtering platform.

The filtering process of the initial design scheme for axial flow compressor based on DDPG is shown in Figure 4.

### 4.2. Optimization Results

A neural network is built with Pytorch 2.4.0, and Adam algorithm is used as the optimization algorithm. The learning rate of the policy network is $1\times 10^{-4}$ and the learning rate of the value network is $1.5\times 10^{-4}$. The prioritized replay buffer capacity is $1\times 10^{5}$, the minimum training set batch is 500, the number of training episodes is 1000, and the maximum number of steps per episode is 100. Target efficiency is set at 84.5% and target surge margin is set at 10%.

**Figure 5:**
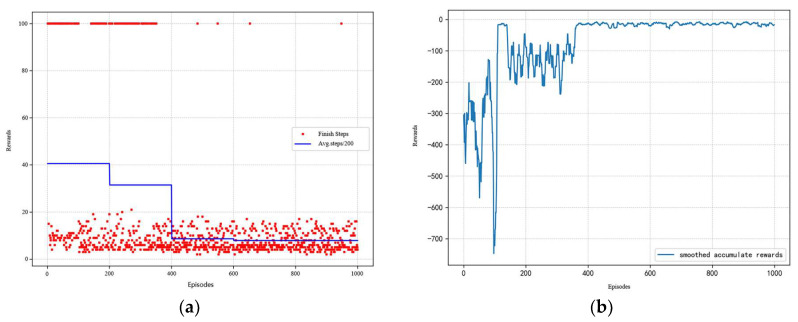
Training results of DDPG. (**a**) The steps of each episode; (**b**) the reward of each episode.

The training history of the DDPG for the initial design of a six-stage axial compressor is given in Figure 5. At the beginning of training (about the first 400 rounds), almost every training round requires the complete execution of 100 steps. It indicates that the agent has not yet mastered an effective design policy and needs to accumulate experience through a lot of exploration. As the training process advances, the system shows significant learning effects. The number of steps required to reach the design goal shows a monotonically decreasing trend, which indicates that the agent has gradually mastered a more efficient design policy. The cumulative reward value continues to climb, which reflects that the quality of the design policy is continuously improving.

**Figure 6:**
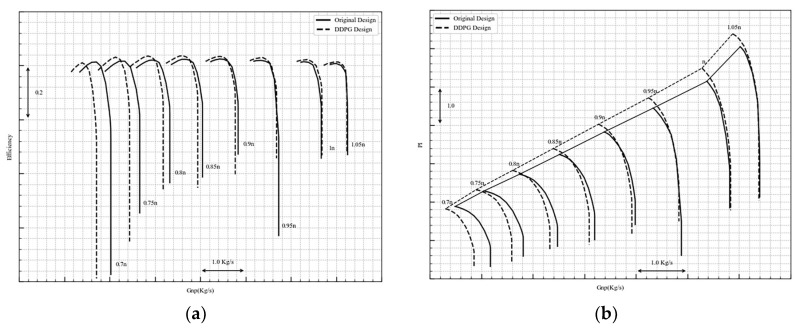
Optimization comparison. (**a**) Efficiency characteristics; (**b**) pressure ratio characteristics.

HARIKA can calculate the characteristics of compressor at different rotating speeds. An optimization comparison of the characteristic lines is given in Figure 6, in which the original design is further optimized by DDPG, resulting in an increase in both efficiency and surge margin. Figure 6a shows the variation in the efficiency characteristic lines; the maximum efficiency is increased from 83.75% to 84.65% at 100% rotating speed. The overall trend of the characteristic line is upward, indicating that operating of the compressor has gained varying degrees of improvement in efficiency. Figure 6b is the pressure ratio characteristic. After the DDPG optimization the pressure ratio at all speeds is significantly improved, indicating that a larger surge margin than the original is obtained after the optimization.

**Figure 7:**
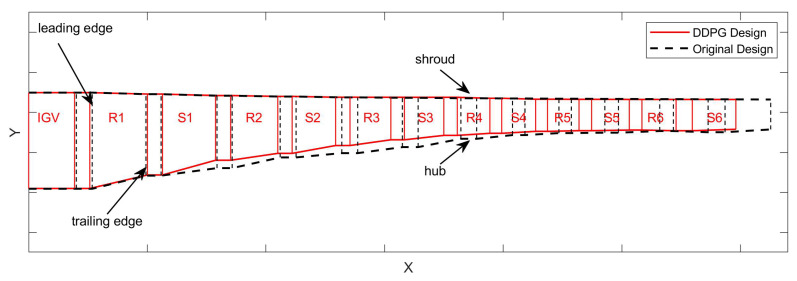
Optimization comparison of flow path.

With satisfying the design requirements, the flow path length is shortened by 4.7%. The blade height is basically the same as before and the intermediate section is reduced by 13.74%. A comparison of the flow path with optimization is displayed in Figure 7.

**Figure 8:**
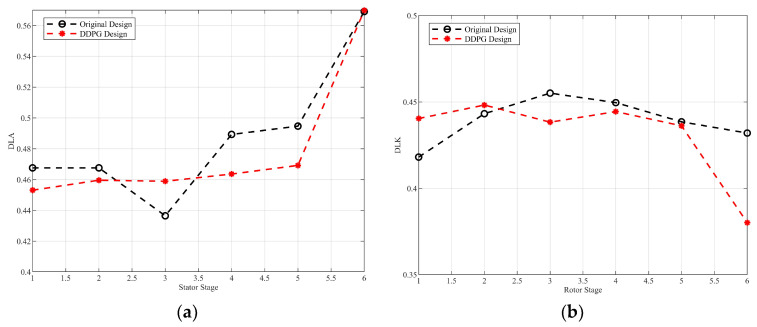
Comparison of diffusion factors. (**a**) Stator diffusion factor; (**b**) rotor diffusion factor.

The diffusion factor (DF) evaluates the diffusion degree of the airflow in the channel. Specifically, the diffusion factor can be divided into two interrelated components. The rotor diffusion factor (DLK) characterizes the relationship between the pressure increase and the kinetic energy loss in the boundary layer. When it exceeds the critical threshold, the adverse pressure gradient will lead to the separation of the boundary layer flow. The stator diffusion factor (DLA) embodies the load distribution on the airflow. Excessive load is prone to inducing secondary flow phenomena such as complex flow separation vortices. The comparison of diffusion factors by optimization is indicated in Figure 8.

After optimizing the parameters of the multistage compressor by DDPG, the simulation results show that the diffusion factors at all levels show a significant reduction. It means the aerodynamic expansion characteristics of the blade channel are improved, and the distribution of the static pressure gradient in the flow channel is smoother. Meanwhile, the stability of the boundary layer is enhanced, which effectively suppresses the flow separation. Furthermore, the distribution of the blade load is more reasonable, and the intensity of the circumferential secondary flow is weakened. Overall, the systematic reduction in the diffusion factor marks the overall optimization of the flow structure of the compressor and a significant improvement in the flow stability, which provides an important theoretical basis and optimization direction for the aerodynamic design of the highly loaded compressor.

With the exception of the inlet tip diameter of rotors, the specific values of design variables with optimization are given in Table 2.

## 5. Model Explanation and Rule Extraction

### 5.1. SHAP Analysis of Key Design Parameters on Compressor Performance

**Figure 9:**
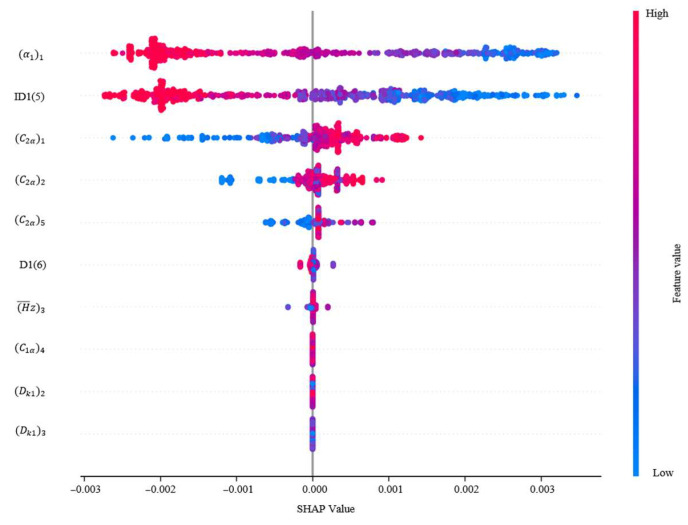
SHAP density map for efficiency.

**Figure 10:**
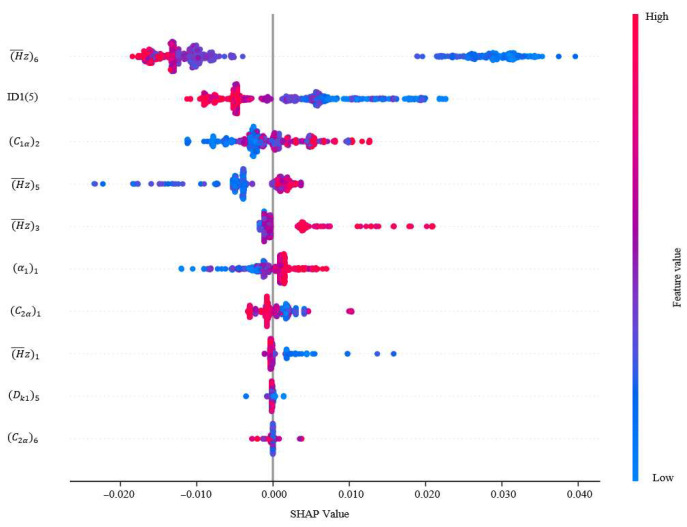
SHAP density map for surge margin.

The SHAP method is used to calculate the Shapley value of each variable to quantify its contribution to the compressor performance, as shown in Figure 9 and Figure 10. Each row of the plot represents a feature, the horizontal coordinate represents the SHAP value, and the color represents the value of the feature. A spot represents a sample. The wider region means there are a large number of samples gathered. As the samples scatter, it means the feature has a greater impact on the target. If the value of SHAP is positive, the current feature has a promoting effect on the performance. If the value of SHAP is negative, it indicates that the current feature has an inhibiting effect on the performance. The variables have been normalized in order to eliminate the effect of different magnitudes on the performance of the compressors.

Figure 9 and Figure 10 represent the characteristic density plots for compressor efficiency and surge margin, respectively. It can be seen from Figure 9 that higher values of reaction and inlet airflow angle negatively affect the compressor efficiency, while the values of axial velocity have the opposite effect. In Figure 10, higher values of reaction also have a negative gain on the surge margin, while high values of inlet airflow angle have a positive gain on the surge margin. The effect of inlet airflow angle on performance is more complex. High airflow angle values have a positive impact on efficiency, while exhibiting completely opposite effects on surge margin. But the magnitude of the effect on the surge margin is smaller than that on the efficiency.

**Figure 11:**
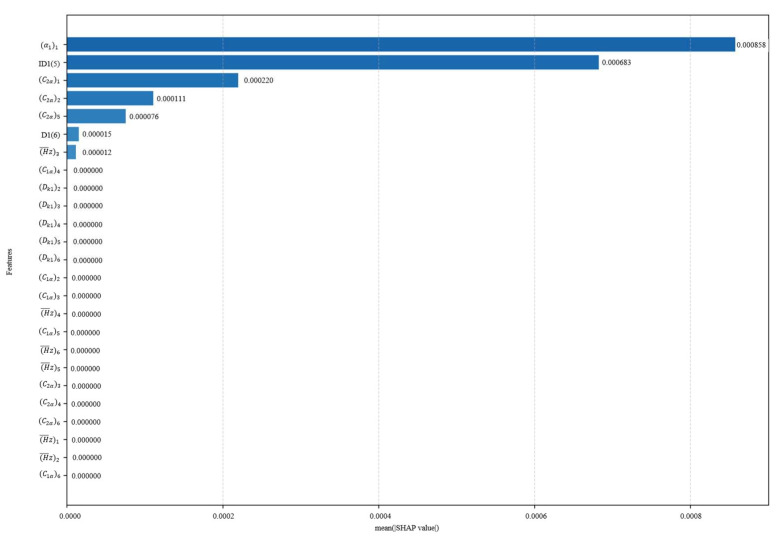
Ranking chart of the importance of features for efficiency.

**Figure 12:**
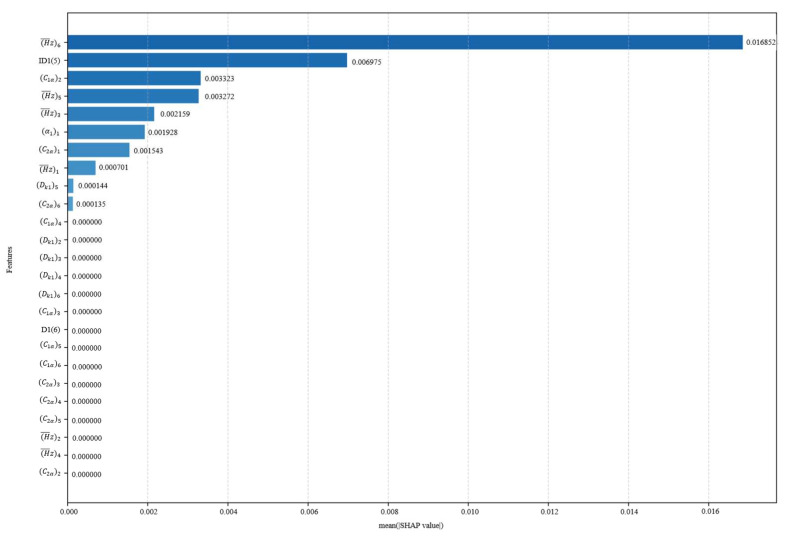
Ranking chart of the importance of features for surge margin.

The SHAP scatter plot further shows the direction of influence of each variable. If the eigenvalue and the corresponding SHAP value are positive, it means that the increase in the variable contributes to the improvement of efficiency and surge margin. Considering the degree of influence of each design variable on the performance, the order of importance is shown in Figure 11 and Figure 12. The relative contribution of each variable to the efficiency as well as margin is visualized by the average of the absolute Shapley values normalizing to sum to 100%. In the whole study of design variables, the inlet flow angle and reaction have a greater influence on the efficiency, contributing 43.44% and 34.58% to the model output, respectively. And the reaction is an important driver of the margin variation with a contribution of 18.84%.

In summary, to obtain a balance between efficiency and surge margin, it is necessary to focus on the values of key variables such as inlet flow angle, reaction, etc. The SHAP enhances the interpretability of the reinforcement learning model and provides a scientific basis for subsequent model optimization and rule extraction.

### 5.2. Decision Tree Rule Extraction for Initial Design

The goal of this work is to improve the explainability of reinforcement learning. In this section, the general architecture for extracting design rules from deep reinforcement learning is presented, as well as a detailed explanation of the design policy acquisition and the decision tree extraction.

Decision trees are regarded as a technique for transport and explainable machine learning, which is capable of generating policies by learning compact representations of relations. Therefore, decision trees can be used to extract implicit knowledge from trained deep reinforcement learning networks.

Largely due to the powerful representational learning capabilities of deep neural networks, reinforcement learning has been successfully applied to the initial design of aero-engine, which can deal with highly nonlinear and complex relationships. However, the implicit behavior of deep neural networks is not known to humans, which creates a significant barrier to understanding the decision-making process and key feature information influencing factors. One of the fundamental problems is that the neural network is a black box and the knowledge in the structure is implicit, which prevents understanding of how it makes decisions.

**Figure 13:**
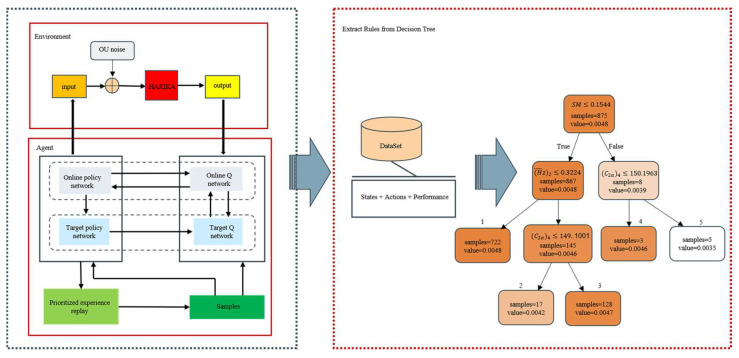
Deep network extraction decision tree architecture.

Aiming to understand how DNNs make decisions, they are only considered high-quality trained policies if they perform well with minimal fluctuations. They then capture the implicit knowledge of deep reinforcement learning models through these quality policies, as indicated in Figure 13.

In this paper, the DDPG is employed to generate the initial design policy for compressor. As it is given a state, it can query the design policy to obtain the corresponding action. Ideally, it should be recognized whether the obtained policy captures the implicit knowledge in the design system. The distribution of actions generated by the design policy is almost similar to the optimal distribution and can achieve high cumulative rewards without divergence. In the initial design of DDPG, the extraction of optimal actions after policy convergence is the central aspect of constructing interpretable rules. During 1000 training rounds the state–action combinations that meet the design requirements after algorithm convergence are collected as well as the performance data under the corresponding states. By adopting a constrained action space processing method, such as physical feasible tailoring, it guarantees the engineering realizability of the generated actions, which form the core data base for extracting the design rules.

To ensure the quality of the extracted rules, these state–action pairs that satisfy the design requirements are selected as the training data for the decision tree. The state–action pair of the trajectories is adopted directly to mimic the high-quality policies, focusing only on the mapping between the inputs and the outputs of the executed actions without considering the internal structure of the source policies.

The CART regression tree is used to fit the collected data. With following the black-winged kite algorithm for automatic optimization, the maximum depth is set to 4 and the minimum number of samples is set to 3. According to the SHAP analysis in the previous section, the reaction has an important effect on the performance of compressor. Since the SHAP reflects the global effect, the specific changes in reaction may be different for different compressor states in the local policy. Now the design variable of reaction is taken as an example to describe design rules extracted. The concise design rules are stored in Table 3, with the maximum step of each adjustment action set as 0.005.

**Figure 14:**
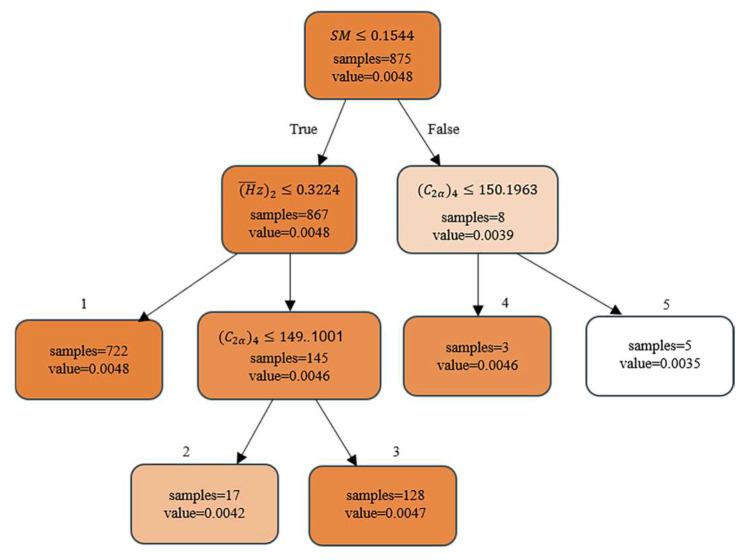
Reaction design rule decision tree.

Value represents the relative change in reaction at the maximum adjustment step, and the DDPG algorithm follows the above five design rules in the adjustment of reaction. It can balance the performance and constraints of compressor and obtain higher rewards. The rules in the decision tree are displayed in Figure 14.

The decision tree is split at different nodes in different stages, reflecting the dynamic change in feature importance at different stages. CART splits the nodes by recursively selecting the current optimal features. And the “optimal” at each step is based on the mean square error of the current dataset. The color shades represent the proportion of samples. The left subtree is less than or equal to the threshold, and the right subtree is greater than the threshold. The root node splitting condition is SM≤0.1544, which indicates that the DDPG primary consideration is the current state of the surge margin (e.g., whether it is close to the surge boundary) when deciding how to change the reaction. The adjustment of reaction strength needs to obey the stability demand in priority. Due to the small number of samples with high margin, it may cause the rule to be non-robust. So it should focus on the second-layer nodes. The split conditions of the second-layer nodes are ${\left(\bar{H}z\right)}_{2}\leq 0.3224$ and ${\left(C_{2\alpha }\right)}_{4}\leq 150.1963$, which indicate that the focus of compressor design is different when the size of surge margin is different. The third layer is subdivided into two branches through the sub-node ${\left(C_{2\alpha }\right)}_{4}\leq$ 149.1001. The leaf node represents the final decision. For example, if SM≤0.1544 and ${\left(\bar{H}z\right)}_{2}\leq$ 0.3224, then value = 0.0048. Here, the final value takes the average of the samples.

The selection of decision tree nodes has stage dependence, and shallow nodes tend to choose features that have a significant impact on the overall data variance, such as surge margins. Deeper nodes deal with residuals or nonlinear relationships and may choose interaction features or noise. The specific analysis of the rules in Table 3 is as follows.

Rule 1: When the surge margin is insufficient, the flow stability becomes the primary optimization objective. At the same time, if the load factor of second stage is low, it means that the rotor is not capable of conducting enough work on the flow. This may cause the kinetic energy of the airflow to decay in the stator channel, which, in turn, triggers flow separation. In this case, the accelerating effect of the rotor on the airflow can be effectively enhanced by substantially increasing the reaction, increasing the pressure ratio of the rotor section. Thus, reducing the diffusion load of the stator can be reduced by suppressing the boundary layer separation.

Rule 2: System stability is challenged when compressor is operated with insufficient surge margin. The high load factor indicates that the rotor is already at a high load state. An excessive increase in reaction may trigger leakage flow in the tip and separation at the boundary layer, leading to a significant increase in the risk of stall. At the same time, the lower axial velocity of the intermediate stage means that the kinetic energy of the airflow through the stator channel is insufficient. In this complex case, the use of moderate reaction regulation improves the stator flow conditions by appropriately increasing the rotor loading and avoids the stall problem due to over-regulation. This policy fully considers the interaction of stage matching.

Rule 3: In the case of a low surge margin and high rotor load, although the rotor itself has already borne a large load, the higher axial velocity of the intermediate stage indicates that the flow in the stator has sufficient kinetic energy reserve. By significantly increasing the contribution of the rotor to the pressure rise of the stage, the stage load distribution can be optimized. At the same time, the diffusion of the high axial velocity is utilized to compensate for the possible rise in stator load caused by the increase in reaction. By comprehensively considering the actual flow capacity of the stator, a better stage matching is realized under the premise of ensuring stability.

Rule 4: When the compressor is operating in a high surge margin region, the system has a sufficient stability margin. The focus of design optimization can be shifted to the improvement of aerodynamic efficiency. However, the low axial velocity of the intermediate stage indicates that the kinetic energy of the flow in the stator is relatively insufficient, and there is a potential risk of diffusion loss. By appropriately increasing the rotor load, the stage energy distribution can be optimized to effectively reduce diffusion losses due to low axial velocity while ensuring that no significant flow separation occurs in the stator. This makes full use of the design freedom provided by the high stability margin to pursue the optimization of aerodynamic performance.

Rule 5: When the compressor is in the high stability operating range and the axial velocity is significantly increased, the high velocity flow in the stator has excellent diffusion capabilities. A strong kinetic energy reserve can suppress the risk of flow separation. Under these ideal conditions, the use of minimum necessary reaction regulation demonstrates the design concept of performance optimization. By precisely controlling the rotor load increment, unnecessary flow losses are minimized while ensuring stage matching.

By establishing a hierarchical conditional judgment architecture, the design rule deeply associates the reaction regulation with compressor stability index, rotor load, and stator flow characteristics. It is not only in line with the basic theory of mechanical aerodynamic design but also fully reflects the intelligent decision-making advantage of deep reinforcement learning algorithms in multi-objective optimization problem.

### 5.3. Rule Validation

For the same problem, in the same environment and the same initial state, experimental tests are conducted to validate the performance of the design rules. The size of cumulative rewards obtained under the guidance of the DDPG and decision tree rules is compared for a total of 500 training rounds. The rule set is hard-coded with if–then logic as the control policy, which directly maps states to actions. Meanwhile the DDPG policy adjusts the network parameters through online learning. The two methods share the same reward function to ensure the fairness of the performance comparison.

**Figure 15:**
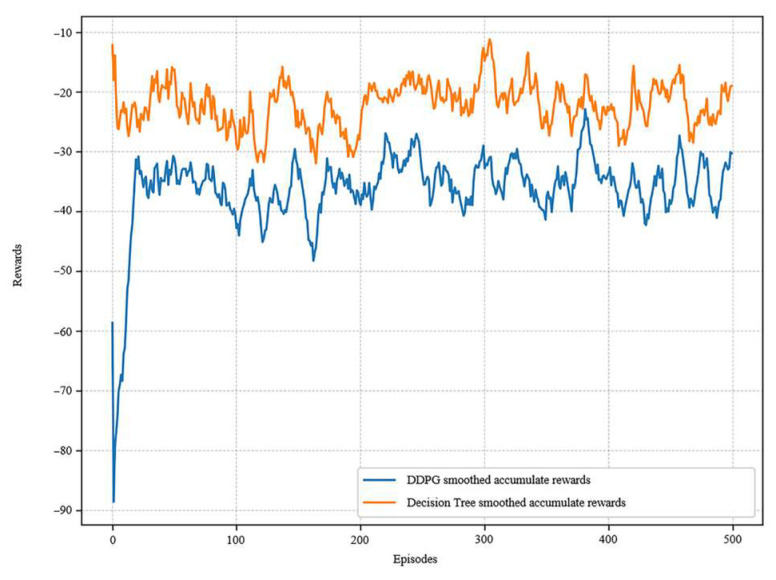
Rewards comparison of DDPG and decision tree.

The rules extracted from the decision tree are able to obtain high rewards from the beginning in Figure 15, showing an improvement compared to the DDPG. It reveals that the rules extracted from the decision tree maintain the performance of the deep reinforcement learning network and have a good generalization ability.

## 6. Discussion

The core contribution of this study is to extend the optimization of reinforcement learning applied to the initial design of compressor, while using a decision tree algorithm to extract interpretable design rules from DDPG policies. Decision trees have the advantages of simple structure and high interpretability.

There are potential limitations to the current study. First, further attention can be paid to the synergistic effects among design variables, combined with interpretable techniques such as the attention mechanism, to deepen the understanding of the decision-making process of reinforcement learning. Second, the current method is based on a one-dimensional analysis procedure and, in the future, Computational Fluid Dynamics (CFD) simulation or data-driven agent models can be introduced to improve the optimization accuracy while ensuring computational efficiency.

In addition, the current extracted rule values are sample averages and the accuracy can be improved in the future. Random Forest or Gradient Boosting Tree can be attempted instead of a single decision tree to capture the subtle changes in the continuous movements. In the short term, this method can be used for initial compressor design selection. And, in the long term, it can assist in accomplishing the full life cycle design of the compressor. This also has potential applications in the design of energy equipment with high reliability requirements.

## 7. Conclusions

In this paper, based on the DDPG, intelligent filtering of the initial optimized design scheme of an axial-flow compressor is successfully realized. The results show that the optimized design scheme achieves an efficiency of 84.65%, which is 0.9% higher than the original design. Meanwhile, the surge margin reaches 10.75%, which is 8.39% higher than the baseline scheme. Compared with the traditional design, the reinforcement learning can automatically acquire design experience through interaction with the environment, providing support for the intelligent compressor design.

This study quantitatively evaluates the effect of each design variable on the performance of compressor. It specifies the direction of gain in the performance due to design parameters based on the positive and negative characteristics of the SHAP values. From the global view, the values of inlet airflow angle and reaction are inversely proportional to the efficiency magnitude of the compressor. In terms of surge margin, it obtains a larger surge margin by appropriately reducing the load factor and reaction of the last stage. And it is beneficial for surge margin by increasing the load factor of the intermediate stage, the axial velocity at the inlet of the second stage rotor, and the airflow angle. The present algorithm clarifies the feature-driven decision-making in reinforcement learning and provides a guide to the rule extraction for the subsequent design.

The initial design rule with clear physical meaning is extracted from the DDPG-generated policies using a decision tree algorithm with high interpretability. To validate the effectiveness of the design rule, this study designs 500 rounds of comparison experiments. The results show that the rule is able to maintain the optimization performance of the reinforcement learning network. This study not only expands the range of optimization variables for reinforcement learning in the initial optimal design of compressor but also establishes a transformation method from black-box models to interpretable rules, which provides a research idea for constructing more transparent and interpretable models.

## Figures and Tables

**Table 1:** The definitions and ranges of design variables.

Definition	Design Variable	Ranges
Inlet airflow angle	${{(\alpha }_{1})}_{1}$ (°)	55–70
Inlet tip diameter of rotor stage i (i=2,…,6)	${\left(D_{K1}\right)}_{i}$ (m)	0.40–0.43
Axial velocity of rotor inlet of stage i (i=2,…,6)	${\left(C_{1\alpha }\right)}_{i}$ (m/s)	110–170
Axial velocity of rotor outlet of stage i (i=1,…,6)	${\left(C_{2\alpha }\right)}_{i}$ (m/s)	110–170
Load factor for stage i(i=1,…,6)	${\left(\bar{H}z\right)}_{i}$	0.2–0.35
Reaction	ID1(5)	0.5–0.8
Reaction increment	D1(6)	0–0.025

**Table 2:** The optimization results of DDPG.

Design Variables	Original Design	DDPG Design
${{(\alpha }_{1})}_{1}$ (°)	73	65.71
${\left(C_{1\alpha }\right)}_{2}$ (m/s)	136.8	153.52
${\left(C_{1\alpha }\right)}_{3}$ (m/s)	136.8	157.54
${\left(C_{1\alpha }\right)}_{4}$ (m/s)	151.3	157.11
${\left(C_{1\alpha }\right)}_{5}$ (m/s)	143.7	151.46
${\left(C_{1\alpha }\right)}_{6}$ (m/s)	129.5	132.83
${\left(C_{2\alpha }\right)}_{1}$ (m/s)	130.9	134.36
${\left(C_{2\alpha }\right)}_{2}$ (m/s)	134.9	145.34
${\left(C_{2\alpha }\right)}_{3}$ (m/s)	132.7	151.83
${\left(C_{2\alpha }\right)}_{4}$ (m/s)	146.3	148.24
${\left(C_{2\alpha }\right)}_{5}$ (m/s)	130	137.1
${\left(C_{2\alpha }\right)}_{6}$ (m/s)	113	118.54
${\left(\bar{H}z\right)}_{1}$	0.25	0.2807
${\left(\bar{H}z\right)}_{2}$	0.28	0.3076
${\left(\bar{H}z\right)}_{3}$	0.27	0.3055
${\left(\bar{H}z\right)}_{4}$	0.27	0.2932
${\left(\bar{H}z\right)}_{5}$	0.24	0.2711
${\left(\bar{H}z\right)}_{6}$	0.23	0.2195
ID1(5)	0.62	0.6654
D1(6)	0.05	0.0212

**Table 3:** Reaction design rules.

Design Rules
① IF SM≤0.1544$\;\&\;{\left(\bar{H}z\right)}_{2}\leq 0.3224\;\;$THEN value = 0.0048
② IF SM≤0.1544$\;\&\;{\left(\bar{H}z\right)}_{2}\leq 0.3224\;$$\;\&\;{\left(C_{2\alpha }\right)}_{4}\leq 149.1001\;\;$THEN value = 0.0042
③ IF SM≤0.1544 $\&\;{\;(\bar{H}z)}_{2}>0.3224\;$$\;\&\;{\left(C_{2\alpha }\right)}_{4}>149.1001\;\;$THEN value = 0.0047
④ IF SM>0.1544$\;\&\;{\left(C_{2\alpha }\right)}_{4}\leq 149.1001\;\;$THEN value = 0.0046
⑤ IF SM>0.1544 $\;\&\;{\left(C_{2\alpha }\right)}_{4}$ > 150.1963 THEN value = 0.0035

## Data Availability

Data are contained within the article.
